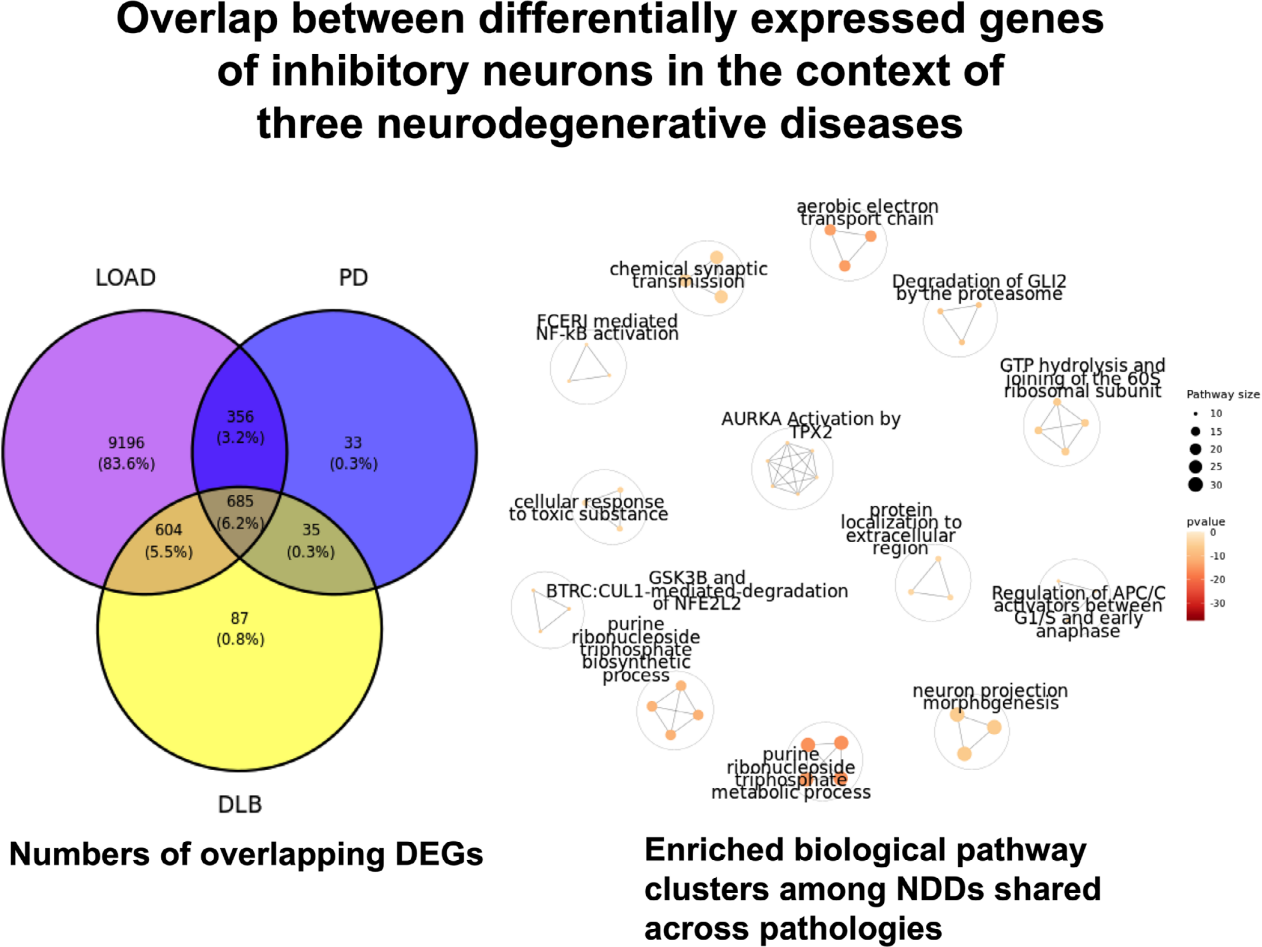# Single‐cell transcriptomic landscapes of neurodegenerative diseases: towards mapping shared and distinct mechanisms

**DOI:** 10.1002/alz.088856

**Published:** 2025-01-09

**Authors:** Elliot Keats Shwab, Zhaohui Man, Daniel Gingerich, Melanie E Garrett, Gregory Crawford, Allison Ashley‐Koch, Ornit Chiba‐Falek

**Affiliations:** ^1^ Duke University School of Medicine, Durham, NC USA

## Abstract

**Background:**

Age‐related neurodegenerative disorders (NDDs) continuum includes late‐onset Alzheimer’s disease (LOAD), Dementia with Lewy bodies (DLB), and Parkinson’s disease (PD) exhibit shared and distinct clinicopathological characteristics. Each of the different NDDs is characterized by a complex genetic etiology and although numerous loci have been identified via GWAS, and the causal genes and the specific neuronal and glial cell subtypes through which they exert their pathogenic effects are yet to be fully elucidated. We aimed to untangle the genetic complexity of NDDs, and to identify shared and distinct biological pathways and disease driver cell‐subtypes across NDDs.

**Method:**

Single‐cell transcriptomic profiling was performed using LOAD, DLB, PD and normal brains (12/group). Differential expression was analyzed using the single‐nucleus (sn)RNA‐seq datasets by Nebula. To investigate shared biological pathways, we analyzed the common DEGs across NDD pathologies for each cell‐type using Metascape and aPEAR. To identify distinct DEGs and biological pathways we comparted directly between each two pathologies. The vulnerable and disease driver cell‐subtypes were characterized by beta‐regression and AUCell analyses, respectively.

**Result:**

We identified cell‐subtype specific differential expressed genes (DEGs) for each NDD compared to control. A catalogue of common DEGs across NDDs in each cell type showed the largest number of upregulated‐DEGs in inhibitory neurons (∼700). Pathway analysis using the shared DEGs indicated enrichment for biological pathways including, neurodegeneration, stress response, RNA and protein metabolism, and mitochondria dysfunction. Noteworthy, we observed overlap with pathways detected for NPS comorbidity. Differential gene expression analysis between LOAD vs PD identified >5000 upregulated‐DEGs in microglia followed by Astrocytes and OPC and found enrichment for pathways including oligodendrocytes maturation and myelination, while the majority of upregulated‐DEGs in PD compared to LOAD were identified in oligodendrocytes and enriched in biological terms related to protein biosynthesis and energy metabolism. We showed that microglia and oligodendrocytes cell‐subtypes are drivers of LOAD pathology whereas PD pathogenic effect is driven by excitatory neurons.

**Conclusion:**

This study represents the most comprehensive a systematic interrogation of gene dysregulation in a spectrum of NDDs in an unprecedented cell‐subtype resolution. The results enhance our understanding of the shared and distinct genetic factors and biological processes underlying age related NDDs.